# Associations between health, productive performance and oral fluid biomarkers in commercial pig farms

**DOI:** 10.1186/s40813-024-00418-1

**Published:** 2024-12-31

**Authors:** Mario Andre S. Ornelas, Edgar Garcia Manzanilla, José J. Cerón, Alba Ortín-Bustillo, María José López-Martínez, Carla Correia-Gomes, Finola C. Leonard, Lorena Franco-Martínez

**Affiliations:** 1 Pig Development Department, Animal and Grassland Research and Innovation Centre, Teagasc Moorepark, P61C996, Fermoy, Co. Cork, Ireland; 2 School of Veterinary Medicine, University College Dublin, Belfield, Dublin, Ireland; 3 Interdisciplinary Laboratory of Clinical Analysis, Regional Campus of International Excellence ‘Campus Mare Nostrum’, University of Murcia (Interlab-UMU), University of Murcia, Campus de Espinardo s/n, Murcia, 30100 Spain; 4 Animal Health Ireland, Carrick-On-Shannon, N41 WN27 Ireland

**Keywords:** Analyte, Pig herds, Production and health factors, Saliva, Swine

## Abstract

**Background:**

Oral fluid contains analytes that may be reflective of health and welfare in pig herds. Additionally, oral fluid collection is a more convenient and cost-effective option when compared to blood sampling, increasing the potential of oral fluid as a non-invasive alternative tool. While a growing number of biomarkers can be measured in porcine oral fluid, the use of these analytes to compare commercial herds in veterinary practice is still limited. This study describes associations between oral fluid biomarker measurements and farm indicators of health and performance in 18 commercial farms.

**Results:**

Using principal component analysis, three clusters of farms were identified, differing mostly in weaner and finisher mortality, daily gain and antimicrobial resistance. These groups were then compared in terms of oral fluid biomarker profiles. With regards to farm group (cluster), haptoglobin was higher in pigs from low-performing farms, especially when compared with pigs from high-performing farms (*P* = 0.01). Oxytocin tended to decrease in pigs from high-performing farms to low-performing farms (*P* < 0.10), while procalcitonin tended to be lower in pigs from high-performing farms compared to intermediate-performing farms (*P* = 0.07). Using regression trees, haptoglobin measured in late finishers was associated with weaner and finisher mortality. Further, high creatine kinase and low procalcitonin early after weaning were associated with low piglet mortality, whereas low daily gain was related to high alpha-amylase in late weaners and high creatine kinase in pigs at the start of the finisher stage.

**Conclusions:**

Haptoglobin, procalcitonin, oxytocin, creatine kinase and alpha-amylase, measured in oral fluid, should be further studied as good candidates to assess pig herds and predict performance at a batch level, through a non-invasive approach. Herd performance and health figures at a particular time point are not always available and alternative measures, like oral fluid biomarker results, could be useful to anticipate health and welfare issues and adjust management.

## Background

Oral fluid (OF) is a valuable sample to study pig herds because its composition can reflect the health and physiological status of animals [1–3]. Saliva, oro-naso-pharyngeal secretions and serum transudates make up most of the fluid in the oral cavity [4–6]. As a result, pathogens [7–9], antibodies [10–12], antimicrobials [13–15], hormones, proteins and other biomarkers [16–19] can be detected and measured in OF. While the composition of OF suggests it is suited for a wide range of diagnostic applications, pathogen detection still accounts for the vast majority of oral fluid-based on-farm diagnostics [1].

The use of OF is particularly relevant in the context of pig production because it is a non-invasive alternative to blood collection, it is less stressful, requires less expertise, and obtaining samples is facilitated by the pig’s natural behaviour to explore materials using the mouth [20, 21]. Group-level sampling allows the representation of a population through a single sample, which might increase diagnostic sensitivity compared to other specimens [22], reduce the possible influence of inter-individual variability, and permit earlier detection of health and welfare issues [23]. Other advantages include the reduced cost, time and training required for sample collection, which are of utmost relevance for intensive pig production.

Oral fluid biomarkers have been developed through the use of randomised studies to assess stress and welfare by the measurement of analytes such as cortisol, alpha-amylase or oxytocin [2, 16, 24], immunity and inflammation, through adenosine deaminase (ADA) or haptoglobin [25–27] and oxidative status [28]. Other conditions that can be investigated include sepsis, studied using procalcitonin (PCT) [32], and general homeostasis which can be assessed through creatine kinase (CK), lactate dehydrogenase (LDH) and total protein [18, 29]. Nonetheless, the use of these analytes to assess and benchmark commercial herds in veterinary practice is still limited. Differences in biomarker results according to age [18], sample collection and processing method [30, 31] and health [29, 32] have been described. However, in order to assess the value of OF biomarkers for on-farm diagnostics it is necessary to study how differences in health and welfare status are reflected in terms of biomarker results. In this study, we collected OF samples at different production stages from a cohort of pig farms with different productive performances and of different health status to study the relationships between farm characteristics and biomarker measurements. A profile including analytes of stress and welfare (cortisol, alpha-amylase and oxytocin), inflammation and immunity (ADA and haptoglobin), sepsis (PCT), and general homeostasis (CK, LDH and total protein) was measured in OF samples.

## Methods

### Farm selection

Eighteen Irish farrow-to-finish pig farms were selected to ensure representativeness in terms of health and productive performance. Farm size ranged from 130 to 2400 in number of sows. Performance data were retrieved from the Teagasc Profit Monitor database [33]. Biosecurity scores based on the Biocheck. UGent™ scoring system [34] were obtained from the Animal Health Ireland Pig HealthCheck database [35]. As described by Rodrigues da Costa et al. [36], the Biocheck. UGent™ scoring system consists of a questionnaire that assesses farm management practices, divided in six categories of internal biosecurity and six of external biosecurity. Questions in each category have a fixed score that adds up to 100. Internal and external biosecurity scores are calculated as the weighted average of the scores of the relevant categories, and the average of both is used to obtain overall biosecurity score. Information regarding porcine reproductive and respiratory syndrome (PRRS) status and post-weaning use of zinc oxide and in-feed antimicrobials (ZnOAb) was collected during farm visits. Variables used to characterise farms included pigs/sow/year (number of pigs produced per sow per year), piglet mortality, weaner + finisher mortality (sum of the mortality in the weaner and finisher stages), post-weaning daily gain (g/day), age at sale (days), PRRS status (positive or negative), post-weaning use of zinc oxide and medicated feed (yes or no), internal biosecurity score, external biosecurity score and overall biosecurity score (between 0 and 100).

Farms were described for these variables and further characterized for *Salmonella* prevalence and for antimicrobial resistance (AMR) through the use of *Escherichia coli* (*E. coli)* as an indicator organism. Fluoroquinolone-resistant *E. coli* and extended-spectrum beta-lactamases (ESBL) and AmpC cephalosporinases producing *E. coli* were investigated in samples collected at every stage. “AMR” is used hereafter to refer to the level of fluoroquinolone-resistant, and extended-spectrum beta-lactamases (ESBL) and AmpC cephalosporinases producing *E. coli* detected in this study.

### Sampling stages

Farm visits took place between March and July of 2022. Each farm was visited once to collect samples from pigs at the following production stages: one week after weaning (W1), one week prior to transfer to the finishing facility (W2), one week after transfer to the finishing facility (F1) and one week prior to slaughter (F2). The former three sampling stages capture the effects of weaning and relocation on biomarker results, while stage F2 was chosen for being close to slaughter. Weaning in Irish pig farms normally takes place between 28 and 32 days of age and pigs are moved to the finishing facility at around 12 weeks of age. Slaughter takes place between 22 and 25 weeks of age, when live weights range between 110 and 115 kg.

### Environmental sample collection and pre-processing

A pair of cover-socks (EnviroBootie™, Hardy Diagnostics^®^, California, USA) was used to collect environmental samples at each stage for detection of *Salmonella* and AMR. In an effort to minimize contamination, disposable boot covers were used to cover farm shoe wear prior to putting on the cover-socks. After walking in the pens wearing the cover-socks, these were carefully removed, transferred to a sterile plastic bag and transported to the laboratory under refrigeration for same-day processing. Four socks, one per stage, were processed for each farm. This consisted of transferring 250 ml of buffered peptone water into each bag, followed by hand massaging and incubation at 37 °C for 18 ± 2 h. After incubation, the pre-enriched cultures were aliquoted into sterile 10 ml tube containers for subsequent culturing in selective media.

### Detection of fluoroquinolone-resistant and ESBL-/AmpC-producing E. Coli and Salmonella

The detection of *E. coli* and *Salmonella* spp. isolates was carried out based on the protocols “Isolation of ESBL-, AmpC- and carbapenemase-producing *E. coli* from caecal samples” (EURL-AR, 2019) and ISO 6579-1:2017(E), respectively. For all incubation steps, samples were incubated at 37 °C for 18 ± 2 h. Briefly, for detection of *E. coli* isolates, pre-enrichment samples were first sub-cultured onto MacConkey agar supplemented with cefotaxime (1 mg/L) and ciprofloxacin (1 mg/L). After incubation, presumptive ESBL-/AmpC- producing *E. coli* colonies were sub-cultured onto tryptone bile X-glucuronide (TBX) agar supplemented with cefotaxime (1 mg/L) and/or ciprofloxacin (1 mg/L) agar, respectively, and incubated. For each of the samples from the four stages, presence or absence of antimicrobial resistant isolates was recorded. Accordingly, a farm could have resistance detected on up to 8 plates, if typical growth was present on both cefotaxime and ciprofloxacin supplemented media at all stages. Prevalence of AMR for each farm is reported as the proportion of plates on which resistant isolates were recovered (number of positive plates/8).

For *Salmonella* detection, Modified Semi-solid Rappaport-Vassiliadis (MSRV) agar was inoculated with 100 µl of the pre-enriched culture and incubated. Plates were then inspected for *Salmonella* typical growth, which, if absent, led to a further incubation step of 18 h. Typical growth of *Salmonella* in MSRV agar plates was sub-cultured onto both Xylose Lysine Deoxycholate (XLD) and Brilliant Green agar and incubated. From each plate with suspected *Salmonella* colonies, at least one colony was sub-cultured onto Brilliance™ *Salmonella* and nutrient agar and incubated. If typical growth was observed on Brilliance™ *Salmonella* agar, a small amount of culture from the corresponding nutrient agar plate was used for serological confirmation by slide agglutination testing with polyvalent antisera. For each of the four stages, presence or absence of *Salmonella* isolates was recorded. Prevalence of *Salmonella* for each farm is reported as the proportion of stages positive for *Salmonella* (number of stages positive/4).

### Oral fluid collection and processing

At every stage, oral fluid samples were collected from 3 random pens according to the method described in Ornelas et al. (2023) [37]. Briefly, each sponge was offered to a group of animals to be chewed on until visibly moistened. It was then transferred to a Salivette tube (Sarstedt^®^, Nümbrecht, Germany), centrifuged at 3000 g for 5 min and the resulting supernatant frozen at -20 °C until further analysis.

### Oral fluid biomarker analysis

Biomarker measurements were carried out in 215 samples using the methods described in Ornelas et al. (2023) [37]. Each sample was characterized for a panel comprising analytes of stress and welfare (cortisol, alpha-amylase and oxytocin), inflammation and immunity (ADA and haptoglobin), sepsis (PCT), and general homeostasis (CK, LDH and total protein), all of which were validated for use in porcine OF. In brief, in-house immunological methods utilizing AlphaLISA assays were used for cortisol [38], oxytocin [39], haptoglobin [40] and PCT [32]. For alpha-amylase, ADA, total protein, CK and LDH, commercially available spectrophotometric assays were used [18]. All assays had inter and intra-assay coefficients of variability lower than 15%. The details regarding the kits and assays used can be found in Contreras-Aguilar et al. (2021) [40].

### Statistical analysis

All data were processed and analysed using R version 4.1.3, including R packages car version 3.1.2, ggplot2 version 3.5.1, r.part version 4.1.16 and rpart.plot version 3.1.2. Alpha level for determination of significance was 0.05 and trends are reported between 0.05 and 0.10. The group of eighteen farms was described for twelve farm characteristics expressed as mean ± standard error, median and range.

In order to assess collinearity and explore associations among farm characteristics and between farm characteristics and biomarker results, Spearman’s rank correlation coefficients were computed. Given the multifactorial nature of animal health and performance in pig farms, a single parameter does not contain enough information to comprehensively classify and compare different herds. Thus, PCA was used in the present study as a method to cluster farms based on several health and productive performance parameters. Out of the twelve farm characteristics used to describe the farms, ten were selected for principal component analysis (PCA). Age at sale and overall biosecurity were excluded as they were strongly related to daily gain and internal/external biosecurity, respectively. This analysis was performed to investigate how the eighteen farms clustered according to their characteristics. The variance explained by the two main components was computed as well as the loadings of each component.

Differences in farm characteristics between each farm group (cluster), were assessed using Kruskal-Wallis tests. Post-hoc pairwise comparisons were carried out using Dunn’s tests. For each biomarker, the distribution of measurements was assessed for normality and a logarithmic transformation was performed for non-normally distributed data. A general linear regression model was computed for each biomarker with sampling stage and farm group (cluster) as predictors. Biomarker results per stage and farm group are reported as mean ± standard error.

Regression trees were computed with selected farm characteristics as response variables and biomarker results per stage as predictors to identify subgroups of farms and estimate cut-off biomarker values. Each tree had one response variable from the eighteen farms (daily gain, piglet mortality and weaner + finisher mortality) and thirty-six predictors (nine biomarkers measured at four stages). A node represents a subgroup of farms. Five was set as the minimum number of farms in a node for a split to exist as well as the minimum number of farms at any terminal node. The complexity parameter was 0.05, meaning that for a split to happen, the regression’s R-squared had to increase by at least 0.05. Each node displays the number of farms included and respective mean value of the response variable.

## Results

### Farm characteristics, AMR and Salmonella prevalence

The characteristics that describe the eighteen farms are summarized in Table 1. Isolates resistant to ciprofloxacin, cefotaxime and both antimicrobials were detected in sixteen, six and six farms, respectively, while *Salmonella* was detected on ten farms.

**Table 1:** Farm characteristics for the 18 herds in the study group

	Mean ± SE	Median (min-max)
Pigs/sow/year	27.9 ± 0.6	27.9 (23–32.3)
Piglet mortality (%)	11.5 ± 0.7	11.3 (5.9–17.2)
Weaner and finisher mortality (%)	5.7 ± 0.6	5.2 (3.3–14.0)
Post-weaning daily gain (grams)	741 ± 17	730 (632–865)
Age at sale (days)	176 ± 3	178 (148–203)
AMR prevalence (%)^1^	45 ± 8	31 (0–100)
*Salmonella* prevalence (%)^1^	35 ± 9	25 (0–100)
Herds positive to PRRS (%)	60 ± 10	100 (0–100)
External biosecurity score	82 ± 2	82 (65–93)
Internal biosecurity score	63 ± 3	62 (28–83)
Overall biosecurity score	73 ± 2	73 (47–87)

AMR: antimicrobial resistance; PRRS: porcine reproductive and respiratory syndrome; SE: standard error; ZnO: zinc oxide. ^1^AMR prevalence was calculated as the proportion of plates on which resistant isolates were recovered (number of positive plates/8), and *Salmonella* prevalence was calculated as the proportion of stages positive for *Salmonella* (number of stages positive/4)

### Correlation analysis of farm characteristics and biomarker results

**Fig. 1:**
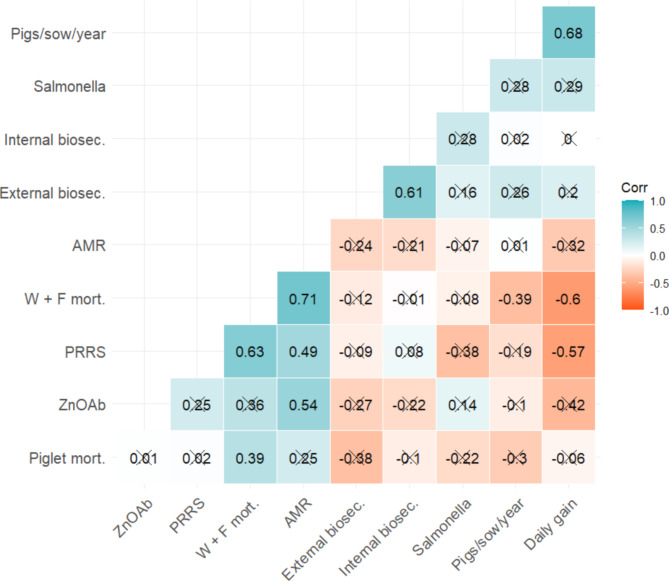
Correlation matrix showing Pearson’s rank correlation coefficients among ten farm characteristics. Cells with an X indicate a non-significant correlation. AMR prevalence was calculated as the proportion of plates on which resistant isolates were recovered (number of positive plates/8), and Salmonella prevalence was calculated as the proportion of stages positive for Salmonella (number of stages positive/4). AMR: antimicrobial resistance prevalence; External biosec.: external biosecurity score; Internal biosec.: internal biosecurity score; Piglet mort.: piglet mortality; Pigs sow year: number of pigs produced per sow per year; PRRS: porcine reproductive and respiratory syndrome status (positive or negative); W + F mort.: weaner + finisher mortality; ZnOAb: use of zinc oxide and medicated feed (yes or no)

Correlation coefficients among farm characteristics are shown in Fig. 1. Positive and negative correlations with a correlation coefficient (r) greater than 0.50 and lower than − 0.50, respectively, are highlighted. Positive correlations were observed between AMR and weaner + finisher mortality, daily gain and pigs/sow/year, weaner + finisher mortality and PRRS status, internal biosecurity and external biosecurity, and between AMR and use of zinc oxide and medicated feed. Negative correlations were observed between daily gain and weaner + finisher mortality, and daily gain and PRRS status. All other correlations were either non-significant or significant but with r outside the aforementioned range. The only significant correlation coefficients between farm characteristics and biomarker results were between piglet mortality and CK (*r* = -0.61), internal biosecurity and total protein (*r* = -0.48), and use of zinc oxide and medicated feed and LDH (*r* = 0.53).

### Clustering of farms

**Fig. 2:**
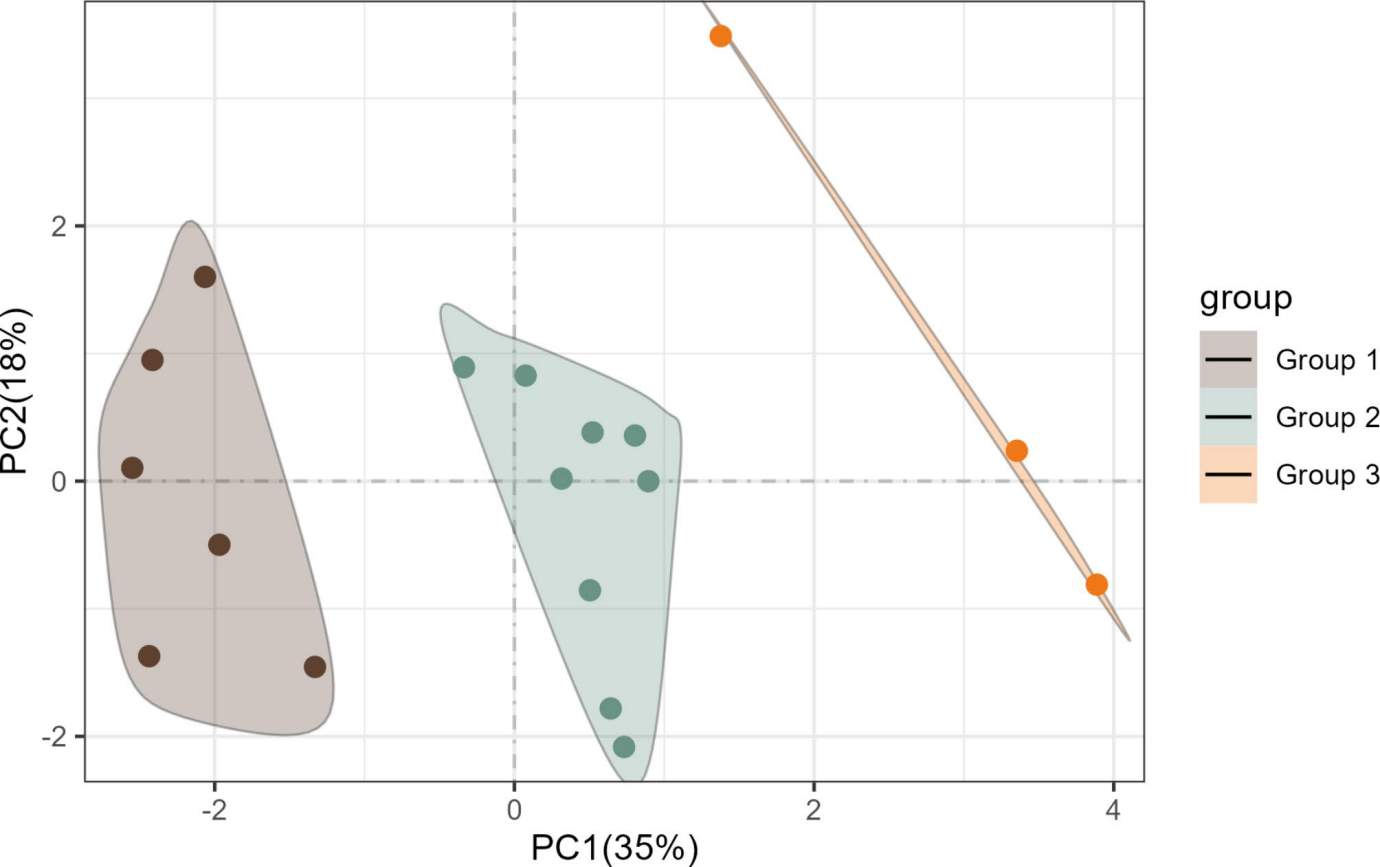
Principal component analysis score plot of ten selected farm characteristics showing 18 farms divided into three clusters. Farm characteristics were pigs/sow/year (number of pigs produced per sow per year), piglet mortality, weaner + finisher mortality (sum of the mortality in the weaner and finisher stages), post-weaning daily gain (g/day), PRRS status (positive or negative), post-weaning use of zinc oxide and medicated feed (yes or no), internal biosecurity score, external biosecurity score, Salmonella prevalence and antimicrobial resistance (AMR). Weaner + finisher mortality, daily gain and AMR were the most important contributors for the first component, whereas internal and external biosecurity scores were the main contributors for the second component

Principal component analysis of ten farm characteristics, including AMR and *Salmonella* prevalence, revealed three clusters of farms containing six, nine and three farms each (Fig. 2). The two main orthogonal contributors explained 35% and 18% of the variance in the data, respectively. The loadings for the first component indicated that weaner + finisher mortality, daily gain and AMR were the most important contributors, whereas internal and external biosecurity were the main contributors for the second component.

**Fig. 3:**
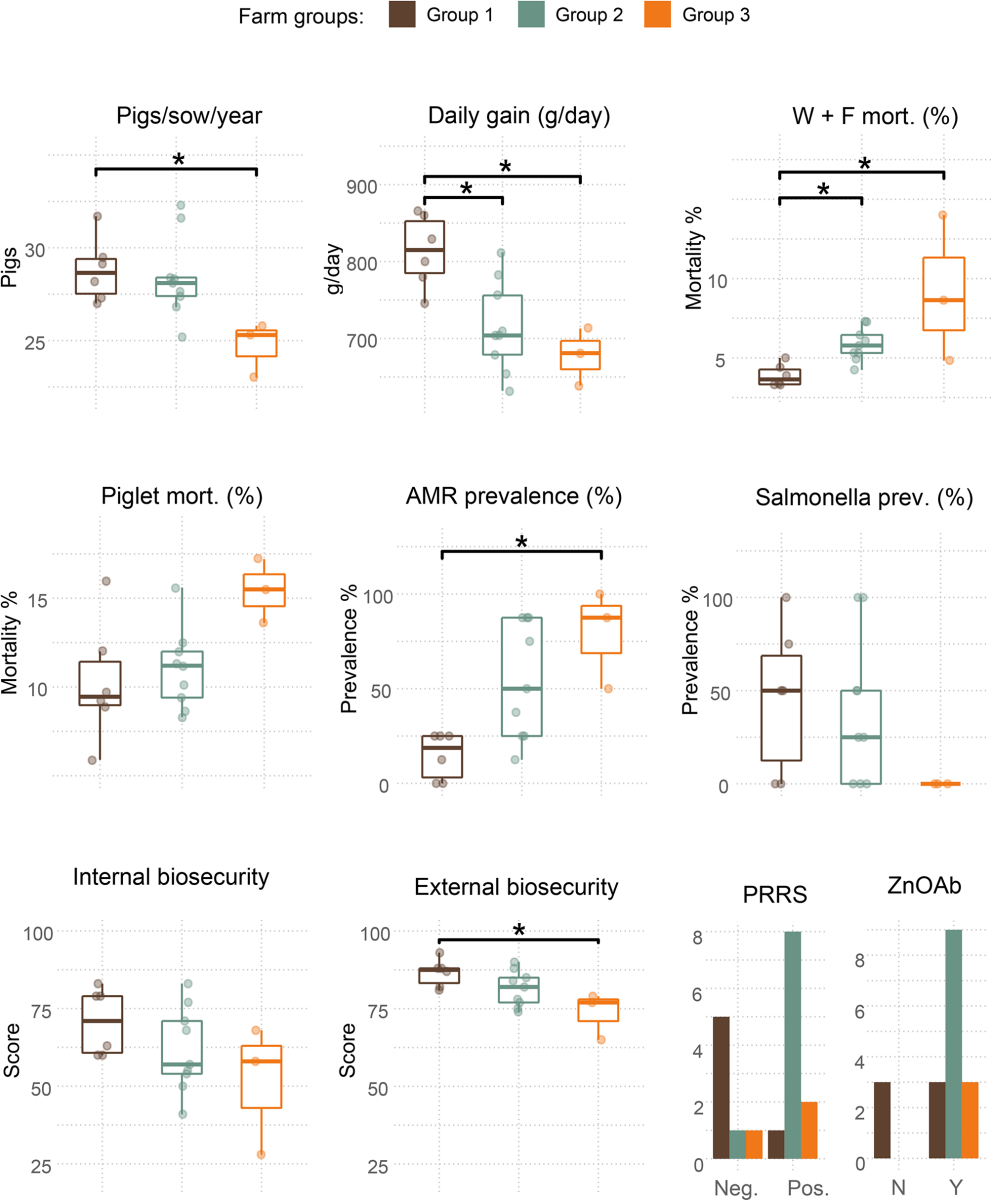
Comparison of farm characteristics between the three farm groups obtained by PCA. Brackets with an asterisk on top indicate a significant difference between two groups. If brackets are absent, the difference was non-significant. Bar plots with PRRS and ZnOAb indicate the number of farms positive and negative to PRRS, and using ZnOAb or not. AMR: antimicrobial resistance; mort.: mortality; N: no; Neg.: negative; Pos.: positive; prev.: prevalence; PRRS: porcine reproductive and respiratory syndrome status; W + F mort.: sum of the mortality in the weaner and finisher stages; Y: yes; ZnOAb: use of zinc oxide and medicated feed. Farm groups are 1: high-performing farms, 2: intermediate-performing farms and 3: low-performing farms

The differences between the three farm groups defined by PCA according to farm characteristics are shown in Fig. 3. Pairwise differences were observed for pigs/sow/year, daily gain, weaner + finisher mortality, AMR prevalence and external biosecurity. Farms in group 1 had the highest mean values for pigs/sow/year, daily gain, external biosecurity, internal biosecurity and *Salmonella* prevalence, all of which had the lowest means for farms in group 3. On the other hand, the means of weaner + finisher mortality, piglet mortality and AMR prevalence were highest for farms in group 3 and lowest for farms in group 1. Based on these results and according to the benchmarking criteria used in the national databases Teagasc Profit Monitor [33] and Pig HealthCheck [35], farms in group 1 were considered high-performing farms and farms in group 3 were considered low-performing farms. Group 2 included intermediate-performing farms, which were similar to group 1 for variables like pigs/sow/year or external biosecurity and to group 3 in variables like daily gain or weaner + finisher mortality. Group 2 also had a higher mean prevalence of PRRS positive herds (0.9 ± 0.1) compared to groups 1 (0.2 ± 0.2) and 2 (0.7 ± 0.3). All farms in groups 2 and 3 used zinc oxide and medicated feed, which was true of only half of the farms in group 1.

### Biomarker results

The results of the nine studied biomarkers by sampling stage and farm group are shown in Table 2. Stage affected the values of all biomarkers, while farm group only showed differences for haptoglobin and trends for cortisol, oxytocin and PCT. No interactions were found between stage and group. Stage W1 had the highest results of all stages for all biomarkers. On the other hand, the lowest biomarker measurements were mostly in stage F2. When considering biomarker results according to farm group, haptoglobin was higher for group 3 compared to group 1 (*P* = 0.012). Further, groups 1 and 2 tended to have higher cortisol levels (*P* = 0.060) than group 3. Oxytocin showed a trend to increase from group 1 to group 3 (*P* = 0.086) and PCT tended to have higher values for group 2 compared to group 1 (*P* = 0.07).

**Table 2:** Means of oral fluid biomarker measurements at four production stages according to farm group

Biomarker	Group	Stage					*P*-value (SEM)		
		W1	W2	F1	F2	All	Stage	Group	Int
**Cortisol** **ng/mL**	1	65.2	45.9	39.2	52.7	50.8			
	2	84.6	51.9	30.8	39.9	51.8	< 0.001	0.060	0.616
	3	62.9	39.0	12.8	11.8	31.6	(6.8)	(5.4)	(10.7)
	All	70.9^a^	45.6^b^	27.6^b^	34.8^b^				
**Alpha**-a**mylase** **IU/mL**	1	1.76	0.61	0.99	0.36	0.93			
	2	1.97	1.41	1.62	0.94	1.49	< 0.001	0.188	0.253
	3	3.50	0.91	0.37	0.34	1.28	(0.30)	(0.23)	(0.47)
		2.41^a^	0.98^b^	1.00^b^	0.55^b^				
**Oxytocin** **ng/dL**	1	58.4	32.0	23.3	21.8	33.9			
	2	59.9	50.3	37.5	34.5	45.5	0.019	0.086	0.581
	3	60.4	41.5	70.2	35.2	51.8	(6.7)	(5.1)	(10.2)
		59.6^a^	41.3^ab^	43.7^ab^	30.5^b^				
**Haptoglobin** **µg/mL**	1	2.22	1.63	1.05	0.35	1.31^b^			
	2	2.70	1.52	1.54	0.64	1.60^ab^	< 0.001	0.012	0.282
	3	3.08	1.29	2.15	1.26	1.94ª	(0.16)	(0.12)	(0.24)
		2.66^a^	1.48^b^	1.58^b^	0.75^c^				
**PCT** **µg/mL**	1	1.77	1.15	0.71	0.77	1.10			
	2	2.75	0.99	0.96	1.21	1.48	< 0.001	0.070	0.305
	3	1.90	1.20	0.88	0.86	1.21	(0.17)	(0.13)	(0.26)
		2.14^a^	1.11^b^	0.85^b^	0.95^b^				
**ADA** **IU/mL**	1	3.26	1.75	1.90	1.08	2.00			
	2	3.93	1.74	1.56	1.24	2.12	< 0.001	0.603	0.195
	3	4.60	1.72	1.40	1.23	2.24	(0.18)	(0.14)	(0.28)
		3.93^a^	1.73^b^	1.62^b^	1.18^b^				
**CK** **IU/L**	1	18.2	9.6	11.1	3.7	10.6			
	2	22.4	9.0	9.4	6.2	11.7	< 0.001	< 0.269	0.238
	3	14.0	10.5	7.9	5.1	9.4	(1.2)	(0.9)	(1.9)
		18.2^a^	9.7^b^	9.5^b^	5.0^c^				
**LDH** **IU/L**	1	111.0	24.2	14.9	9.7	39.9			
	2	110.3	28.5	21.8	15.6	44.1	< 0.001	0.323	0.162
	3	64.9	13.2	32.1	19.0	32.3	(6.2)	(4.9)	(9.7)
		95.4^a^	21.9^b^	22.9^b^	14.7^b^				
**Protein** **mg/dL**	1	75.8	46.9	35.5	36.0	48.5			
	2	92.7	33.2	33.6	29.4	47.2	< 0.001	0.767	0.124
	3	61.8	36.5	39.4	37.9	43.9	(4.7)	(3.7)	(7.3)
		76.7^a^	38.9^b^	36.2^b^	34.4^b^				

Note: Means without a common letter in the same row represent differences between stages, while means without a common letter in the same column represent differences between farm groups. ADA: Adenosine deaminase; CK: Creatine kinase; LDH: Lactate dehydrogenase; PCT: Procalcitonin; W1: one week after weaning; W2: one week before being transferred to finisher facility; F1: one week after being transferred to the finishing facility; F2: one week before slaughter; Int.: interaction; SEM: standard error of the mean. Farm groups are 1: high-performing farms, 2: intermediate-performing farms and 3: low-performing farms

### Regression trees

**Fig. 4:**
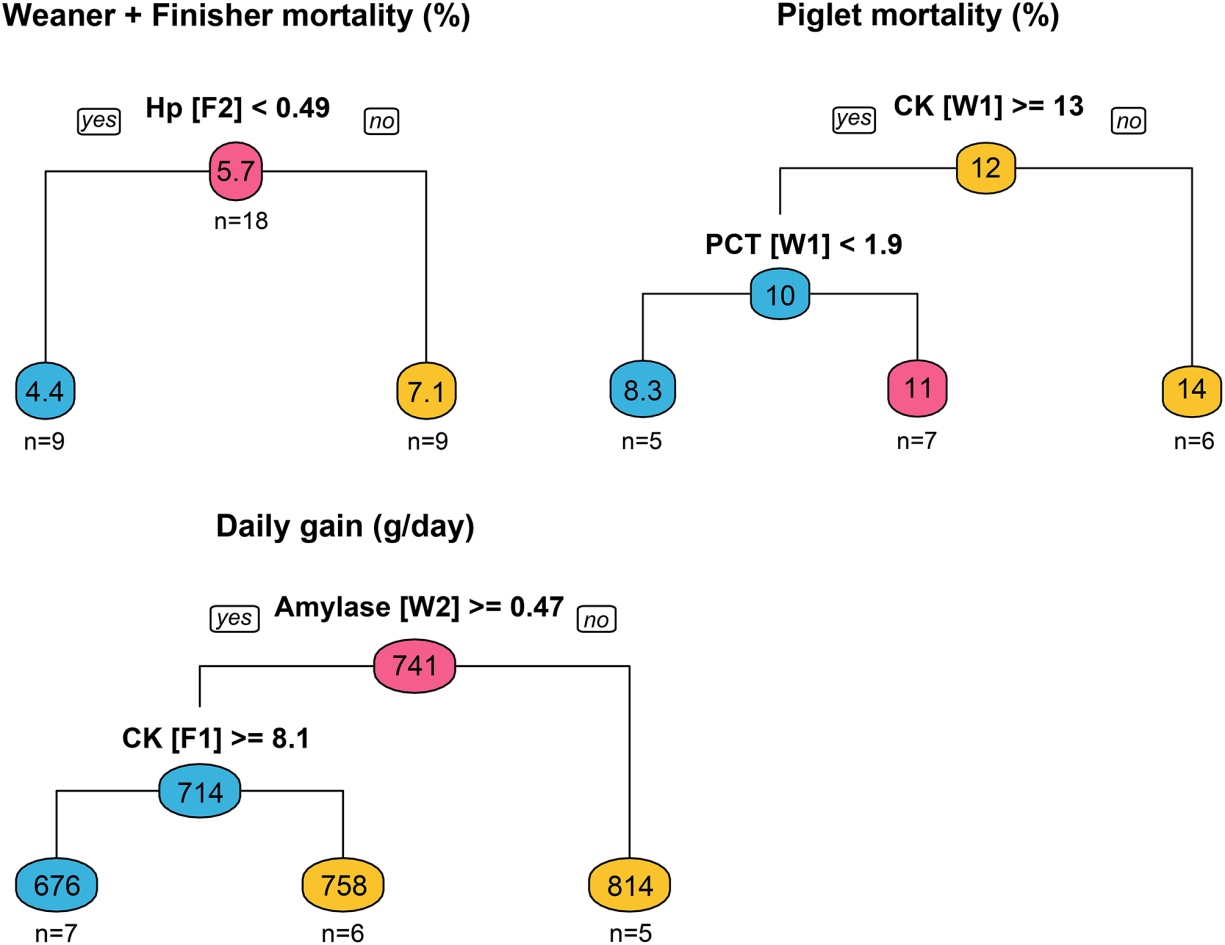
Regression trees for three selected farm characteristics using biomarker results per stage as predictors. Each node represents a sub-group of farms and indicates the mean value of the farm characteristic and the number of farms in the sub-group. Biomarker results per stage are expressed as mean measurements followed by stage in square brackets. W1: one week after weaning; W2: one week before being transferred to finisher facility; F1: one week after being transferred to the finishing facility; F2: one week before slaughter; CK: creatine kinase; Hp: haptoglobin; PCT: procalcitonin

The regression tree for weaner + finisher mortality divided farms in 2 groups, with 4.4% and 7.1% mortality, based on a haptoglobin threshold of 491 µg/mL at stage F2 (Fig. 4). This threshold was determined as the optimal point of splitting the 18 farms, while minimizing the variance of weaner + finisher mortality. For piglet mortality, the regression tree used CK (threshold: 13 IU/L) and PCT (threshold: 1893 ng/mL), both measured at W1, to classify farms in three groups. The same number of groups were obtained by the tree for daily gain, based on results of amylase at W2 (threshold: 475 IU/L) and CK measured at F1 (threshold: 8.1 IU/L).

## Discussion

Several assays have been validated in OF for pigs to measure biomarkers of stress, inflammation, or immune status [2, 18, 24]. However, the use of these biomarkers to assess herd status or predict performance of different batches of pigs is very limited. This study explores the use of biomarkers in OF to assess the health status and productivity of pig herds. A cross-sectional sampling of 18 farms with different health status and productive performance was carried out to study the associations between these farm characteristics and the levels of OF biomarkers. Two different approaches were used to achieve this objective. First, we classified the farms in groups (high-performing, intermediate-performing and low-performing) according to their health and productive performance using PCA, and compared the biomarker results between these groups. Second, we used biomarker results to define thresholds to classify farms based on their main performance variables using regression trees.

Twelve parameters were used to characterize the farms, of which the three main contributors to separate the farms according to the PCA were weaner + finisher mortality, daily gain and AMR, the later probably as a proxy for antimicrobial use. Three farm groups were defined that could be called high-performing, intermediate-performing and low-performing in terms of productive performance and health status. As expected, high-performing farms had high productive performance, low mortality and these were associated with lower AMR and higher biosecurity. On the other hand, low-performing farms had low productive performance, high mortality and were associated with higher AMR and lower biosecurity scores. A third group of farms with characteristics in between high and low performing farms was also defined, and thus designated intermediate-performing. Similar groups were found previously by Rodrigues-Costa et al. when studying biosecurity in Irish pig farms [36]. An unexpected result of our study was the high prevalence of *Salmonella spp* at high-performing farms and absence thereof at low-performing farms. These high-performing farms had higher scores for internal biosecurity than low-performing farms, including cleaning, disinfection and batch management practices, which are known to influence *Salmonella* persistence in the environment. However, it is worth mentioning that there are several risk factors associated with *Salmonella* in pig farms which have not been addressed in this study, such as feeding practices, gastrointestinal disease incidence or vaccination. Since this study was carried out, a new questionnaire specific for *Salmonella* risk factors has been introduced as part of the Animal Health Ireland Pig HealthCheck programme [35] and these risk factors will be considered in the future. It is also possible that the relatively low number of farms in the low-performing group, may have been a contributing factor for this result.

Stage affected all biomarkers whereas no interactions between stage and farm group were present. The highest levels were observed at stage W1 for all biomarkers, which is consistent with the results of Ortín-Bustillo et al. (2022) where animals were sampled at similar ages as those in this study [18]. On the other hand, the decrease in biomarker measurements with age was more evident in the current study.

While the reasons behind the higher biomarker measurements of weaned pigs require further study, we hypothesise that such biomarker profile may reflect the stressful nature of weaning. The more pronounced decrease in biomarker measurements in the present study compared to previous studies may be related to the fact that this study was conducted in commercial farms and previous studies were carried out in experimental farms. In commercial farms, the effects of weaning may be more severe than in experimental farms.

When comparing the farm groups for their biomarkers, haptoglobin showed clear differences, being higher for low-performing farms than for high-performing or intermediate-performing farms, and cortisol, oxytocin and PCT showed trends between farm groups. Haptoglobin is an acute phase protein that has been previously used in different areas of pig health and welfare. It increases as a result of inflammation and infectious diseases like those caused by *Streptococcus suis* [41] or *E. coli* [29]. It therefore makes sense that haptoglobin levels were higher in low-performing farms. Thus, haptoglobin is a solid OF biomarker that should be further characterised for variability between batches within farms and for the particular time frame when it has predictive use. The combination of haptoglobin with other OF biomarkers in the same analysis could also increase its value as a herd health biomarker. Oxytocin has a role in immune response modulation as well as anti-inflammatory and pro-immune adaptive functions [42, 43]. For example, oxytocin increases in early stages of sepsis to limit sepsis-associated organ damage [44]. The increase in oxytocin in pigs from farms with sub-optimal health could indicate a compensatory effect of the organism to cope with inflammation and excessive activation of the immune system. Procalcitonin also tended to be higher in low-performing farms and it is associated with both experimentally induced or naturally occurring septic conditions [32]. Procalcitonin is probably the fastest growing biomarker in human health. Thus, more efforts are needed to describe the levels of oxytocin and procalcitonin and especially to explore their potential use in combination with haptoglobin to obtain better clinical information.

Using the regression tree approach, some of the results were consistent with the differences between farm groups obtained with PCA although new findings were also observed. Haptoglobin was again a clear biomarker related to weaner + finisher mortality separating farms into two groups of high (around 7.1%) and low mortality (around 4.4%). It is worth mentioning that the value of haptoglobin to assess the status of the herd would be limited, once we have the value for mortality, which is available for most pig farms. On the other hand, this analyte was measured at the end of the finisher stage (F2), which suggests it could be representative of the animals’ history. However, this limits the predictive value of the measurement. Ideally, a good predictive biomarker would be measured at early stages to predict how a batch will perform thereafter.

Regression trees also showed that high CK and low PCT early after weaning (W1) were associated with low piglet mortality. This is probably reflective of previous issues because piglet mortality takes place in the lactation stage, prior to W1. Thus, the value of these findings to assess herds and predict batch performance is limited too. As a biomarker of sepsis and bacterial infections, the fact that PCT was lowest among farms with higher piglet survival rates was expected. The higher values of CK, however, were unforeseen, given that increases of this enzyme are often associated with muscle damage and disease. However, it is interesting to point out that CK in pigs is usually highest at weaning, as observed in this and other studies [18], and this analyte has been positively correlated with muscle mass [45, 46]. These findings could lead to the hypothesis that high values of CK at weaning with no signs of disease can be an indicator of high health and performance.

Finally, regression trees showed that low daily gain was related to high alpha-amylase in late weaners (W2) and high CK at the start of the finisher stage (F1). Both alpha-amylase and CK are biomarkers commonly used in different species and in humans [18, 47, 48]. The increase in alpha-amylase could result from higher levels of stress, and possibly pain [49], whereas increased CK could indicate muscle damage [50]. The fact that the regression tree indicated these analytes in late weaner (W2) and early finisher (F1) stages makes them valuable, in this case as biomarkers of herd status and batch performance. The daily gain of a herd or batch at a particular time point is not always available and alternative measures, like OF biomarkers, could be useful. For example, knowing the status of batches of pigs before moving them to the finisher stage could help anticipate issues and adjust management.

## Conclusions

Despite the limited number of farms used in this study, haptoglobin, PCT, oxytocin, CK and alpha-amylase, measured in OF, showed potential to be used to assess pig herd status and predict productive performance. This is a promising result for the future use of porcine OF biomarkers in veterinary medicine. The next steps should be to carry out field studies including a higher number of farms and studying variability between batches within farms. Biomarkers measured late in the production cycle have limited predictive value and some biomarkers showed contradictory results compared to previous studies. These aspects should be considered as limitations that need to be solved to optimise the use of OF biomarkers in pig health management.

## Data Availability

The datasets used in this study are available from the corresponding author upon reasonable request.

## Abbreviations

ADA: Adenosine deaminase
AMR: Antimicrobial resistance
BC_SUBEX: External biosecurity score
BC_SUBIN: Internal biosecurity score
CK: Creatine kinase
ESBL: Extended-spectrum beta-lactamases
F1: One week after transfer to the finishing facility: F2:One week prior to slaughter
Hp: Haptoglobin
LDH: Lactate dehydrogenase
MSRV: Modified Semi-solid Rappaport-Vassiliadis
N: No
Neg.: Negative
OF: Oral fluid
PCA: Principal component analysis
PCT: Procalcitonin
Pigs/sow/year: Number of pigs produced per sow per year
Pos.: Positive
PRRS: Porcine reproductive and respiratory syndrome
SE: Standard error
SEM: Standard error of the mean
TBX: Tryptone bile X-glucuronide
W1: One week after weaning
W2: One week prior to transfer to the finishing facility
W.F_mort: Weaner + finisher mortality
XLD: Xylose lysine deoxycholate
Y: Yes
ZnOAb: Post-weaning use of zinc oxide and in-feed antimicrobials
ZnO: Zinc oxide
